# Determinants of international variation in the prevalence of disabling wrist and hand pain

**DOI:** 10.1186/s12891-019-2791-x

**Published:** 2019-09-18

**Authors:** David Coggon, Georgia Ntani, Karen Walker-Bone, Vanda E. Felli, Florencia Harari, Lope H. Barrero, Sarah A. Felknor, Marianela Rojas, Anna Cattrell, Consol Serra, Rossana Borchini, Eleni Solidaki, Eda Merisalu, Rima R. Habib, Farideh Sadeghian, M. Masood Kadir, Roshini J. Peiris-John, Ko Matsudaira, Busisiwe Nyantumbu-Mkhize, Helen L. Kelsall, Helen Harcombe

**Affiliations:** 1 Medical Research Council Lifecourse Epidemiology Unit, University of Southampton, Southampton, UK; 2 Arthritis Research UK/MRC Centre for Musculoskeletal Health and Work, University of Southampton, Southampton, UK; 3 School of Nursing, University of São Paulo, São Paulo, Brazil; 4 Corporación para el Desarrollo de la Producción y el Medio Ambiente Laboral – IFA (Institute for the Development of Production and the Work Environment), Quito, Ecuador; 5 Department of Industrial Engineering, School of Engineering, Pontificia Universidad Javeriana, Bogotá, Colombia; 6 Southwest Center for Occupational and Environmental Health, The University of Texas Health Science Center at Houston School of Public Health, Houston, TX USA; 7 Center for Disease Control and Prevention/National Institute for Occupational Safety and Health, Atlanta, USA; 8 Program Health, Work and Environment in Central America, Institute for Studies on Toxic Substances (IRET), National University of Costa Rica, Heredia, Costa Rica; 9 North East London NHS Foundation Trust, Goodmayes Hospital, Ilford, Essex, UK; 10 Center for Research in Occupational Health (CiSAL), Universitat Pompeu Fabra, Barcelona, Spain; 11 CIBER of Epidemiology and Public Health, Barcelona, Spain; 12 IMIM (Hospital del Mar Research Institute), Barcelona, Spain; 13 Occupational Health Service, Parc de Salut MAR, Barcelona, Spain; 14 EPIMED Research Center, University of Insubria, Varese, Italy; 15 Department of Social Medicine, Medical School, University of Crete, Heraklion, Greece; 16 Institute of Technology, Estonian University of Life Sciences, Tartu, Estonia; 17 Department of Environmental Health, Faculty of Health Sciences, American University of Beirut, Beirut, Lebanon; 18 Center for Health Related Social and Behavioral Sciences Research, Shahroud University of Medical Sciences, Shahroud, Iran; 19 Department of Community Health Sciences, Aga Khan University, Karachi, Pakistan; 20 Section of Epidemiology and Biostatistics, School of Population Health, Faculty of Medical and Health Sciences, University of Auckland, Auckland, New Zealand; 21 Department for Medical Research and Management for Musculoskeletal Pain, 22nd Century Medical and Research Center, Faculty of Medicine, The University of Tokyo Hospital, Tokyo, Japan; 22 National Health Laboratory Service, National Institute for Occupational Health, Johannesburg, South Africa; 23 Faculty of Health Sciences, School of Public Health, University of Witwatersrand, Johannesburg, South Africa; 24 Department of Epidemiology and Preventive Medicine, School of Public Health and Preventive Medicine, Monash University, Melbourne, Victoria Australia; 25 Department of Preventive and Social Medicine, University of Otago, Dunedin, New Zealand

**Keywords:** Wrist/hand pain, Geographical variation, Pain propensity, Risk factors

## Abstract

**Background:**

Previous research has indicated that wide international variation in the prevalence of disabling low back pain among working populations is largely driven by factors predisposing to musculoskeletal pain more generally. This paper explores whether the same applies to disabling wrist/hand pain (WHP).

**Methods:**

Using data from the Cultural and Psychosocial Influences on Disability (CUPID) study, we focused on workers from 45 occupational groups (office workers, nurses and other workers) in 18 countries. Among 11,740 participants who completed a baseline questionnaire about musculoskeletal pain and potential risk factors, 9082 (77%) answered a further questionnaire after a mean interval of 14 months, including 1373 (15%) who reported disabling WHP in the month before follow-up. Poisson regression was used to assess associations of this outcome with baseline risk factors, including the number of anatomical sites other than wrist/hand that had been painful in the 12 months before baseline (taken as an index of general propensity to pain).

**Results:**

After allowance for other risk factors, the strongest associations were with general pain propensity (prevalence rate ratio for an index ≥6 vs. 0: 3.6, 95% confidence interval 2.9–4.4), and risk rose progressively as the index increased. The population attributable fraction for a pain propensity index > 0 was 49.4%. The prevalence of disabling WHP by occupational group ranged from 0.3 to 36.2%, and correlated strongly with mean pain propensity index (correlation coefficient 0.86).

**Conclusion:**

Strategies to prevent disability from WHP among working populations should explore ways of reducing general propensity to pain, as well as improving the ergonomics of occupational tasks.

## Introduction

Musculoskeletal pain, particularly in the low back and upper limb, is a major cause of disability in working populations. Preventive strategies in the workplace have focused mainly on ergonomic measures to reduce mechanical loading of tissues, which is thought to have an important role in pathogenesis. For example, wrist/hand pain has been linked with repetitive movements of the hand, including the use of computer keyboards [1–3]. In addition, the occurrence of symptoms is associated with psychological characteristics such as low mood and tendency to somatise [2].

Using longitudinal data from the Cultural and Psychosocial Influences on Disability (CUPID) study, we have shown previously that after allowance for occupation and known psychosocial risk factors, prevalent disabling low back pain (LBP) at follow-up was strongly related to the number of anatomical sites other than low back that individuals had reported as painful at baseline [4]. Moreover, across the 45 occupational groups studied, the mean number of anatomical sites with pain at baseline (again excluding the low back) correlated with the prevalence of disabling LBP at follow-up, and in combination with the other risk factors examined, explained most of its large variation between occupations and countries.

This paper explores the extent to which, within the CUPID dataset, similar relationships can be discerned for disabling wrist/hand pain (WHP). Specifically, we aimed to assess: i) the association of general propensity to pain (characterised in this case by the extent of pain in the past 12 months at anatomical sites other than the wrist/hand) with subsequent one-month prevalence of disabling WHP; ii) how prevalence rate ratios (PRRs) and population attributable fractions (PAFs) compared with those for other risk factors; and iii) the extent to which general pain propensity and other risk factors accounted for variation by occupation and country in the prevalence of disabling WHP. We used a longitudinal design with risk factors assessed at baseline and the outcome of prevalent disabling WHP determined at follow-up, so as to avoid bias from simultaneous reporting of risk factors and outcomes.

## Methods

The methods of the CUPID study have been described in detail elsewhere [5]. Data were collected in two phases. The study initially targeted a total of 21,014 workers from 47 occupational groups distributed across 18 countries (1 to 4 groups per country). Potential participants were identified from employment records or other suitable sampling frames, and comprised office workers who regularly used computers, nurses, and “other workers” (mainly carrying out repetitive manual tasks with their hands or arms – for example, mail sorters). Each subject was asked to complete a baseline questionnaire (either by self-administration or at interview, according to occupational group), and usable responses were obtained from 12,426, giving an overall response rate of 70% (> 80% in 33 occupational groups).

The baseline questionnaire, which is available as supporting information to reference [5], was used to derive all of the personal risk factors that we examined, most of which were specified exactly as in our earlier paper on disabling LBP [4]. Additionally, it collected information on adverse beliefs about arm pain and awareness of someone outside work with WHP. Participants were classed as having adverse beliefs about the work-relatedness of pain in the arm, shoulder or hand if they completely agreed that such pain is commonly caused by work; about its relationship to physical activity if they completely agreed that for someone with such pain, physical activity should be avoided as it might cause harm, and that rest is needed to get better; and about its prognosis if they completely agreed that neglecting such problems can cause serious harm, and completely disagreed that such problems usually get better within 3 months.

Also at baseline, the lead investigator for the study in each country provided information about various group-level variables that might be relevant to musculoskeletal pain and its impacts. These were: the unemployment rate in the community from which the occupational group came; whether it was necessary to pay for primary medical care; and the availability of: pay during sickness absence, financial support for ill-health retirement, social security for long-term unemployment, and compensation for work-related pain in the wrist/hand.

After a mean interval of 14 months (80% between 11.6 and 18.6 months), participants in 45 occupational groups were invited to complete a follow-up questionnaire (again by self-administration or at interview), similar in style but shorter than that used at baseline. Among other things, it asked whether during the past month, they had experienced pain in the wrist/hand area (left, right or both) that had lasted for longer than a day, and if so, whether the pain had made it difficult or impossible to perform one or more of five listed activities (writing; locking and unlocking doors; opening bottles, jars or taps; getting dressed; and doing normal jobs around the house). Those who reported that any of these activities had been made difficult or impossible were classed as having disabling WHP.

Further details of the methods of sampling and data collection, definition and distribution of study variables, and ethical approvals (provided by the relevant research ethics committee or institutional review board in each participating country) can be found in earlier reports [4, 5].

Analysis was carried out with Stata v.12.1 software (Stata Corp LP 2012, Stata Statistical Software: Release 12.1, College Station TX, USA). For each individual, we counted the number (from 0 to 8) of anatomical sites other than wrist/hand that had been reported as painful for a day or longer in the 12 months before baseline – a measure that we termed “pain propensity index”. Simple descriptive statistics were used to summarise the relationship of this index to other personal characteristics assessed at baseline. Next, we applied Poisson regression to assess the relationship of disabling WHP in the month before follow-up to pain propensity index and other personal risk factors ascertained at baseline. Associations were summarised by PRRs with 95% confidence intervals (CIs) based on robust standard errors, and to account for possible clustering, we fitted a random intercept for each occupational group. For risk factors that showed statistically significant associations with disabling WHP (*p* < 0.05), we also estimated PAFs. The PAF indicated the proportion of cases in the study population that would be eliminated if, after adjustment for other risk factors, the prevalence among those exposed to the factor were reduced to that among those unexposed.

As well as examining personal risk factors, we fitted models to explore possible impacts of risk factors operating at occupational group-level. These included the variables on which lead investigators from each country had submitted information, together with the group mean pain propensity index, and the group prevalence of: adverse beliefs about arm pain, knowing someone outside work with wrist-hand pain, and having heard about “RSI” or the equivalent.

Finally, we explored the variation in prevalence of disabling WHP between occupational groups, and the extent to which it might be explained by differences in pain propensity and in other risk factors. As well as a simple scatter plot, we calculated ratios of the numbers of cases by occupational group to the numbers that would have been expected: a) based only on the overall prevalence of disabling WHP in the full study sample; b) calculated from a Poisson regression model that adjusted for pain propensity index (using predicted probabilities generated by Stata); and c) calculated from a final Poisson regression model that included all statistically significant risk factors. The dispersions of these ratios across occupational groups were summarised by their geometric standard deviations (SDs). To test whether there was unexplained residual variation in prevalence once all of the measured risk factors had been taken into account, we compared the geometric SD of the ratios derived from the final Poisson regression model with the distribution that would have been expected from random sampling variation. The latter was determined from multiple random simulations in which it was assumed that each individual’s probability of disabling WHP was that which would have been predicted from the final Poisson regression model given his/her exposure to risk factors.

## Results

Within the 45 occupational groups that were included in the longitudinal component of the CUPID study, 11,740 participants provided complete information at baseline about the number of anatomical sites other than wrist/hand, which had been painful in the past 12 months. Of these, 9082 (77%) (3099 men and 5983 women) satisfactorily answered the questions about disabling WHP in the past month at follow-up, and were included in the analysis for this report. Follow-up was 100% for the 3170 participants with pain propensity index > 2 at baseline, as compared with 68% among those with an index of 0 and 69% in those with an index of 1 or 2.

As in our earlier study of LBP, which used a slightly different measure of pain propensity (number of anatomical sites other than low back that were painful in the 12 months before baseline) [4], higher pain propensity was observed in women, at older ages, and among those with low mood and tendency to somatise (data available on request).

A total of 1373 participants (15%) reported disabling WHP in the month before follow-up, and Table 1 summarises its associations with personal risk factors ascertained at baseline. The risk estimates presented were derived from a single Poisson regression model, and thus are mutually adjusted. Clear positive associations were observed with female sex (PRR: 1.7, 95%CI 1.5–2.1), older age (PRR: for age 50–59 vs. 20–29 years 1.3, 95%CI 1.0–1.7), prolonged use of a keyboard or other repetitive movements of the wrist/hand in an average working day (PRR: 1.3, 95%CI 1.1–1.6), and somatising tendency (PRR for ≥2 vs. 0 distressing symptoms: 1.4, 95%CI 1.2–1.6). However, after allowance for these and the other risk factors in Table 1, the strongest associations were with pain propensity (PRR for an index ≥6 vs. 0: 3.6, 95%CI 2.9–4.4), and risk rose progressively as the pain propensity index increased. In contrast, no statistically significant associations were observed with any of the group-level risk factors when they were examined in further models that adjusted for individual-level risk factors (data available on request).

**Table 1:** Risk factors at baseline for disabling wrist/hand pain in past month at follow-up

Risk factor	Number of subjects	Number with disabling WHP	Association with disabling wrist/hand pain		
			PRR	(95%CI)^a^	PAF(%)^b^
Sex					
Male	3099	225	1		
Female	5983	1148	1.7	(1.5,2.1)	35.8
Age (years)					
20–29	2094	252	1		
30–39	2920	369	0.9	(0.8,1.1)	
40–49	2607	459	1.2	(1.0,1.4)	
50–59	1461	293	1.3	(1.0,1.7)	5.3
Smoking status					
Never smoked	5865	913	1		
Ex-smoker	1290	207	1.2	(1.0,1.3)	2.0
Current smoker	1903	251	1.1	(1.0,1.3)	
Missing	24	2			
Use of keyboard or other repeated movements of wrist/hand for > 4 h	6742	1159	1.3	(1.1,1.6)	21.3
Psychosocial aspects of work					
Work for > 50 h per week	2052	178	1.0	(0.8,1.1)	
Time pressure at work	6776	1074	1.1	(1.0,1.2)	8.1
Incentives at work	2509	380	1.1	(1.0,1.2)	
Lack of support at work	2340	444	1.0	(0.9,1.2)	
Job dissatisfaction	1766	233	1.0	(0.9,1.2)	
Lack of job control	1827	289	1.0	(0.9,1.2)	
Job security^c^	6407	993	1.1	(1.0,1.2)	6.3
Number of distressing somatic symptoms in past week					
0	5444	562	1		
1	1978	360	1.2	(1.1,1.4)	5.1
2+	1610	441	1.4	(1.2,1.6)	9.4
Missing	50	10			
Mental health					
Good	3612	477	1		
Intermediate	2739	407	1.1	(1.0,1.2)	
Poor	2697	486	1.2	(1.0,1.3)	4.9
Missing	34	3			
Adverse health beliefs about arm pain					
Work-relatedness	2746	547	1.1	(1.0,1.2)	
Physical activity	1110	174	1.0	(0.9,1.1)	
Prognosis	929	204	1.2	(1.1,1.4)	2.8
Individual pain propensity index					
0	2028	115	1		
1	2014	193	1.4	(1.1,1.8)	4.3
2	1870	262	1.8	(1.5,2.2)	8.7
3	1307	242	2.2	(1.8,2.7)	9.6
4	938	210	2.4	(2.0,2.9)	8.9
5	499	171	3.2	(2.5,4.0)	8.5
6+	426	180	3.6	(2.9,4.4)	9.4
Heard of “RSI” or equivalent	5084	789	1.0	(0.9,1.1)	

^a^Prevalence rate ratio with 95% confidence interval

^b^Population attributable fraction per cent (presented only for associations that were statistically significant (p < 0.05)

^c^Note that because risk was higher in participants who reported their employment as being more secure, insecure employment was taken as the reference for the risk estimate

Table 1 also gives PAF estimates (again adjusted for other covariates) for the personal risk factors which exhibited significant (*p* < 0.05) associations with disabling WHP. The highest PAFs were for a pain propensity index > 0 (49.4%), female sex (35.8%), prolonged use of a keyboard or other repetitive movements at work (21.3%), and report of at least one distressing somatic symptom in the past week (14.4%).

**Fig. 1:**
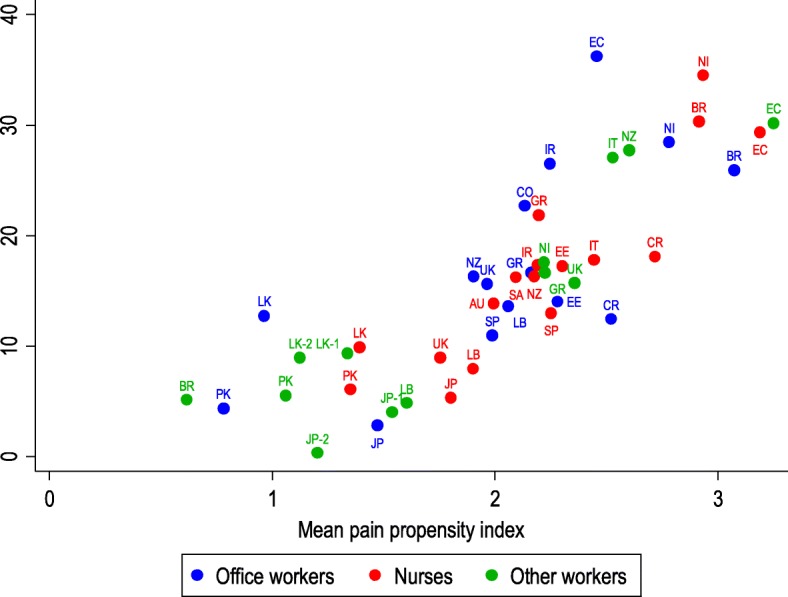
Mean pain propensity index at baseline and one-month prevalence of disabling wrist/hand pain at follow-up by occupational group. Key to countries: AU Australia; BR Brazil; CO Colombia; CR Costa Rica; EC Ecuador; EE Estonia; GR Greece; IR Iran; IT Italy; JP Japan; LB Lebanon; LK Sri Lanka; NI Nicaragua; NZ New Zealand; PK Pakistan; SA South Africa; SP Spain; UK United Kingdom

Figure 1 plots the prevalence of disabling WHP by occupational group in the month before follow-up against the mean pain propensity index of the group at baseline. The latter varied from 0.6 in Brazilian sugar cane cutters to 3.3 in manual workers from Ecuador, while the one-month prevalence of disabling WHP ranged from 0.3% in Japanese sales workers to 36.2% in office workers from Ecuador. There was a strong correlation between the two variables (Spearman rank correlation coefficient 0.86).

The geometric SD of the crude prevalence rates of disabling WHP across the 45 occupational groups was 2.32, but reduced to 2.05 when prevalence was adjusted for baseline pain propensity index. With additional adjustment for all of the other personal risk factors in Table 1, the geometric SD of prevalence rates was further reduced to 1.82, but still was higher than the 95th centile of the expected distribution of SDs if all residual variation were attributable entirely to chance (1.64).

## Discussion

Our analysis indicates that after allowance for occupation and known psychosocial causes, risk of disabling WHP within the CUPID study population was strongly driven by unidentified factors that predispose to musculoskeletal pain in general. Moreover, as for LBP, those factors accounted for much of the large variation in its prevalence across the 45 occupational groups studied.

Our measure of pain propensity was similar to that which we employed in our earlier report on LBP [4], except that it was based on anatomical sites other than the wrist and hand (in the earlier investigation LBP was excluded). The exclusion of WHP from the measure ensured that the observed association with subsequent disabling WHP did not simply reflect the well documented tendency for WHP to persist and recur over time [6]. It may be that as for pain at other anatomical sites, WHP tends to be longer lasting or more frequently recurrent in people who are generally prone to musculoskeletal pain [7], but they could also be at higher risk of its first incidence. Either would manifest as an association with period prevalence.

Because the study was limited to employed adults from selected jobs, the findings cannot necessarily be generalised to the wider populations of participating countries. However, there is no obvious reason why the observed associations, and in particular those with pain propensity, should be peculiar to the occupations studied.

Although follow-up was complete for participants who at baseline had reported pain at three or more anatomical sites other than the wrist/hand, response rates were lower among those with pain propensity indices of 1–2 (69%) and 0 (68%). This may in part reflect a greater commitment to the study from those who experienced most pain, which would be understandable. However, the association with subsequent disabling WHP was apparent even among participants with pain propensity indices < 3 (Table 1). Moreover, the differential response would bias associations with higher pain propensity indices only if disabling WHP lowered response rates among participants with limited or no musculoskeletal pain in the 12 months before baseline, but not at all in those with more widespread pain – which seems an improbable scenario.

A more plausible explanation for the association with pain propensity could be variation in participants’ threshold for reporting symptoms and disability. We took care to check the accuracy with which our questionnaire was translated into local languages through independent back-translation, and we based our outcome measure on disability for everyday activities rather than pain per se. Nevertheless, it is possible that some individuals were more willing to admit to health problems, while others, particularly in certain cultural settings, tended not to complain. Such differences in reporting are a challenge in all epidemiological research on pain, since the symptom is subjective and can only be ascertained by self-report. Sickness absence from work might provide a more reliable measure of disability from pain, and will be examined in a future paper.

Alternatively, the variation in our measure of pain propensity could reflect real differences in participants’ experience of pain, either because they differ in their exposure to external factors that cause pain at multiple anatomical sites, or for physiological reasons. For example, some people may be generally more susceptible to musculoskeletal pain because of differences in their central processing of sensory stimuli [8].

It seems unlikely, however, that the association of disabling WHP with report of pain at other sites is explained by shared underlying pathology in peripheral tissues. While pain in the wrist and hand does sometimes arise from disease or injury at other sites in the neck or upper limb, risk of disabling WHP at follow-up increased progressively across the full range of pain propensity indices from zero to 6 or higher (Table 1). Moreover, we have previously found a strong correlation across occupational groups between baseline prevalence rates of disabling WHP and disabling LBP [9], two symptoms that would not normally be expected to result from the same peripheral pathology. It follows that attempts to prevent disability from WHP should not focus exclusively on risk factors specific to the wrist and hand, whether biomechanical (e.g. forceful repetitive movements of the hand) or psychological (e.g. adverse health beliefs about the causes of arm pain).

The associations that we observed with sex, age, somatising tendency and work involving repetitive movements of the wrist or hands are much as would be expected from earlier research [1–3, 10, 11], including other analyses based on data from the CUPID study [12–15]. It is notable, however, that PRRs and PAFs for our measure of pain propensity were much higher than for other risk factors. This and the strong correlation of mean pain propensity index with the prevalence of disabling WHP by occupational group (Spearman rank correlation coefficient = 0.86) points to a need for better understanding of the determinants of pain propensity and why it varies between countries. If general propensity to musculoskeletal pain could be reduced to levels such as we observed among occupational groups from Pakistan and Sri Lanka, it might be possible to augment substantially the impact of ergonomic controls in the workplace of the type that are currently mandated in the European Union [16], which focus largely on reducing mechanical loading of the upper limb.

There could also be benefits from identification of the risk factors that were responsible for the unexplained residual variation between occupational groups in the prevalence of disabling WHP. However, the pattern of variation by occupational group (Fig. 1) does not give any obvious pointer to what those risk factors might be.

## Conclusions

In summary, our analysis confirms that within the CUPID study, disabling WHP was associated with general propensity to pain, relative risks and PAFs being higher than for other known and suspected risk factors. Moreover, differences in general propensity to pain explained much of the variation between occupations and countries in the prevalence of disabling WHP. It follows that strategies to prevent disability from WHP among working populations should explore ways of reducing general propensity to pain, as well as improving the ergonomics of occupational tasks that load the arm mechanically. A first step might be to explore from what age differences between countries in the prevalence of multi-site pain start to appear, whether migrant populations retain the prevalence of their country of birth or acquire that of the country to which they have moved, and whether there are differences according to age at migration.

## Data Availability

The dataset analysed for the current study is available from the corresponding author on reasonable request.

## Abbreviations

CI: Confidence interval
CUPID: Cultural and Psychosocial Influences on Disability
LBP: Low back pain
PAF: Population attributable fraction
PRR: Prevalence rate ratio
SD: Standard deviation
WHP: Wrist/hand pain
